# Temporal changes in intensity of bird parasite infections are dependent on latitude in the Western Palearctic

**DOI:** 10.1371/journal.pone.0346587

**Published:** 2026-04-09

**Authors:** Anders Pape Møller, Santiago Merino, Juan José Soler, Frank Adriaensen, Alejandro Cantarero, Tapio Eeva, Jordi Figuerola, Marina García-del Río, Jorge Garrido-Bautista, Dieter Heylen, Alfonso Marzal, Erik Matthysen, Piotr Matyjasiak, Ana Claudia Norte, Magdalena Ruiz-Rodríguez, Milena Svobodová, Eszter Szöllősi, Janos Török, Francisco Valera, Jesús Veiga, Nadia Ziane

**Affiliations:** 1 Laboratoire d’Ecologie, Systématique et Evolution, CNRS UMR, Université Paris-Sud, Orsay Cedex, France; 2 Department of Evolutionary Ecology, National Museum of Natural Sciences, Spanish Higher Council for Scientific Research (CSIC), Madrid, Spain; 3 Department of Functional and Evolutionary Ecology, Estación Experimental de Zonas Áridas (EEZA-CSIC), Almería, Spain; 4 Evolutionary Ecology Group, University of Antwerp, Wilrijk, Belgium; 5 Department of Physiology, Veterinary School, Complutense University of Madrid, Madrid, Spain; 6 Department of Biology, FI-20014 University of Turku, Finland; 7 Department of Wetland Ecology, Estación Biológica Doñana, CSIC, Sevilla, Spain; 8 CIBER de Epidemiología y Salud Pública, Madrid, Spain; 9 Department of Life Sciences, University of Coimbra, MARE - Marine and Environmental Sciences Centre, Coimbra, Portugal; 10 Department of Zoology, Faculty of Sciences, University of Granada, Granada, Spain; 11 Instituto de Investigación en Recursos Cinegéticos (IREC-CSIC, UCLM, JCCM), Ronda de Toledo 12, Ciudad Real, Spain; 12 Department of Zoology, University of Extremadura, Badajoz, Spain; 13 Institute of Biological Sciences, Cardinal Stefan Wyszyński University in Warsaw, Warsaw, Poland; 14 Department of Parasitology, Charles University, Faculty of Science, Prague, Czechia; 15 Department of Systematic Zoology and Ecology, Behavioural Ecology Group, ELTE, Eötvös Loránd University, Budapest, Hungary; 16 Hungarian Institute for Forensic Sciences (HIFS), Institute of Forensic Genetics, Department of Reference Sample Analysis. Budapest, Mosonyi utca, Hungary; 17 Laboratory of Biosurveillance environmental, Department of Biology, Faculty of Sciences, University of Badji Mokhtar-Annaba, University, Annaba, Algeria; Environmental Research Center (CRE), ALGERIA

## Abstract

In this study we compare the intensity and prevalence of parasites and reproductive parameters across 14 bird populations sampled in two different seasons separated by approximately 10 years apart, in the Western Palearctic, to test for climate-parasite associations. Overall, 9 different bird species and 62 different host-parasite interactions were studied. We found non-significant trends between the two sampling years in terms of reduced clutch and brood size. However, we observed a decrease in population size between the sampling years, while differences in laying date were negatively related to temperature change between the years. Feather parasites and non-dipteran parasites tended to decrease in both prevalence and intensity, while dipteran parasites showed a few changes with time between the two sampling periods. The prevalence of blood parasites showed a non-significant increase between the two years studied. Importantly, the magnitude and even the direction of the temporal changes in parasitism experienced by different host species across populations depended on latitude, with the northernmost populations showing the smallest decrease in parasite intensity. In addition, changes in temperature between the two study periods decreased with latitude. These results, therefore, point to a potential effect of climate change on the incidence of parasitic diseases, but with variable magnitude and direction across a latitudinal gradient in Europe.

## Introduction

Climate change is influencing both parasite and bird populations [1]. For example, climate change has already advanced spring migration, caused changes in birds’ habitat, increased the incidence of disease transmission, advanced egg-laying time, decreased food availability, and provoked declines in bird populations [2]. Concerning parasites, Mennerat et al. [3] and González-Bernardo et al. [4] showed long-term temperature-driven changes in the intensity of parasite infestation by blowfly (*Protocalliphora* spp.) larvae in the nests of Blue tit (*Cyanistes caeruleus*) and Pied flycatcher (*Ficedula hypoleuca*) populations in southern Europe. Likewise, Castaño-Vázquez and Merino [5] and Merino et al. [6] showed differential effects of climatic variables on the incidence of different ectoparasite and blood parasite species in a population of Blue tits in a mountainous area of central Spain across years. These population-specific studies show the flexibility of hosts and parasites in responding to changes in weather conditions over the years. However, alteration of microclimate inside the nest cavity can affect bird and parasite populations, with increasing temperature or humidity often negatively affecting both of them [7,8]. In addition, the magnitude of these effects varies with latitude in different populations of the same bird species [9]. In this sense, the existence of latitudinal gradients in the distribution and incidence of various parasites has been reported in different host taxa [10–14] and in the case of birds, especially for vector-borne blood parasites [15–21]. Environmental conditions are changing unevenly at different latitudes due to climate change, which could result in varying effects on the distribution of parasites and hosts. This, in turn, may influence the spread of certain parasites and their impact on host populations. Therefore, it is important to understand whether bird-parasite interactions are shifting with latitude.

A pivotal study that examined these trends is Møller et al. [22], which analyzed paired data from 89 parasite populations across 24 species of bird hosts in the western Palearctic, with an average interval of 10 years between the first and second time of sampling for each population. The studied parasite taxa included protozoa, feather parasites, dipterans, ticks, mites and fleas. They investigated whether changes in abundance and prevalence of parasites were related to changes in host body condition, reproduction and population size of hosts between the two sampling periods. They reported an increase in parasite abundance over time even though this was not significantly related to change in temperature at the time of breeding within the study sites. However, they found a decrease in host body condition and clutch size with increasing temperature between the first and second period of sampling. Additionally, changes in the parasite abundance were negatively related to changes in clutch size, brood size and body condition of the hosts. Although they did not detect any effects of latitude on changes in temperature, the changes in laying date between the first and second study year were negatively related to latitude.

Here we report a follow-up study, which compares new and historical data on intensity and impact of parasites on their bird hosts, by checking whether the relationships found by Møller et al. [22] have persisted or changed 10 years later. We asked researchers to return to exactly the same bird populations in 2021, and to record parasitological, host demographic and host density data in the same way as in the previous study, to develop a paired design to test for climate-driven change in host-parasite interactions [22]. Within-population comparisons are known to be particularly powerful because they allow the separation of within-population variance from between-population variance [23]. We used the statistical advantage of this approach to explore the effects of climate change across a broad range of species and populations.

The objective of this study was to investigate the relationship between climate change and host-parasite interactions across two sampling periods. Specifically, we tested (1) if prevalence and intensity of parasites, and abundance of hosts have changed over time within study sites; (2) if the changes in parasite prevalence and intensity were related to changes in temperature within study sites; (3) if the changes in prevalence and intensity of parasites were related to changes in body mass of their hosts; (4) if a change in population density of hosts can be predicted by a change in intensity of parasites or by a change in temperature; and (5) if the magnitude of effects varies with latitude across the study sites.

## Materials and methods

### Study populations

**Fig 1:**
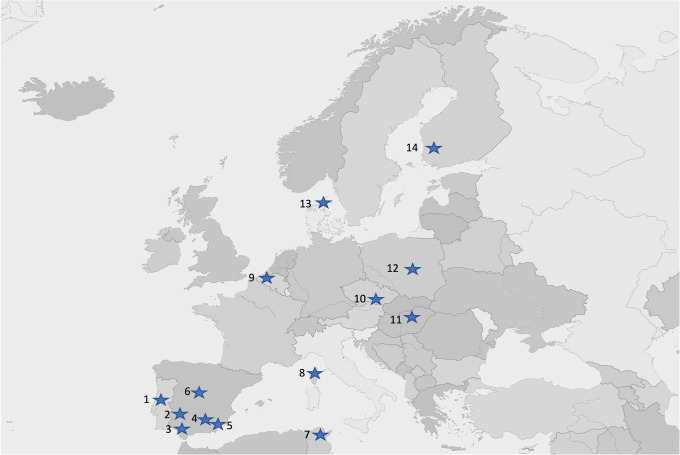
Geographical distribution of the 14 sites for the study of temporal change in intensity and prevalence of parasites of birds. 1. Coimbra (Portugal); 2. Badajoz (Spain); 3. Seville (Spain); 4. Guadix (Spain); 5. Tabernas (Spain); 6. Valsaín (Spain); 7. El Kala (Algeria); 8. Corsica (France); 9. Antwerp (Belgium); 10. Milovice forest (Czechia); 11. Budapest (Hungary); 12. Lomianki (Poland); 13. Kraghede (Denmark); 14. Harjavalta (Finland). See S1 and S2 Tables for more information on species sampled at each locality and Table S3 for references on sampling, identification and quantification methods at each locality. Modified from Mapswire: https://mapswire.com/maps/europe-political-maps/ provided under a Creative Commons (CC-BY 4.0) license.

In February 2021 we requested 59 scientists who had previously published on bird-parasite interactions to participate in the project including all researchers implied in the work by Møller et al [22]. Unfortunately, only a small number of researchers from that previous study were still working on host-parasite interactions. A direct comparison between data collected in 2001 and 2010 with those collected in this second study was not possible; only data from some of the sites investigated in 2010 were used. We also contacted new researchers and disseminated the call for collaboration through the SPI-Birds network (https://spibirds.org/en; [24]) mailing list. Two reminders were sent and eventually 23 scientists from 14 localities (Fig 1) participated in the study, collecting data on 62 different host-parasite interactions for 9 bird species (*Coracias garrulus* from locality 5, *Cyanistes caeruleus* from localities 6, 7, 8 and 11, *Delichon urbica* from locality 2, *Ficedula albicollis* from locality 11, *F. hypoleuca* from localities 6 and 14, *Hirundo rustica* from localities 12 and 13, *Parus major* from localities 1, 9, 10, 11 and 14, *Passer domesticus* from locality 3, and *Pica pica* from locality 4). We deliberately tried to collect all recent samples during the 2021 breeding season, although dates of breeding seasons vary for each species and locality, to reduce potential bias due to interannual variation. However, in one case data were collected in 2020 (Seville) and in two others in 2022 (Granada and Budapest). Although this approach may reduce variation in estimated differences due to the second year being the same for all populations, geographic variation in climatic conditions during 2021 was sufficiently large among study host populations to ensure conclusions that are independent of the particular climatic conditions of 2021. Similarly, although most of the first samples were collected in 2010, in some cases the first sampling was done in 2005 (Budapest and Coimbra), in 2011 (Lomianki), in 2013 (Seville) and in 2015 (Milovice forest) (S1 and S2 Tables) from studies not included in the previous work by Møller et al [22]. Then, since climate and/or hosts’ traits, including parasitism, may be the consequence of yearly trends due to climate change, we explicitly tested for the effect of the number of years spanned between the first and the second study periods on temperature and parasite and host variables, while statistically controlling for the time elapsed between studies.

We asked all participants to use exactly the same methods in the second sampling (2020–22) as during the first (2005–2015), and also that the same person conducted or at least supervised the study, thus ensuring that all studies were consistent in methodology over time to avoid inter-observer variability. That is, each study uses its own methodology to collect, identify and quantify parasites, and the level of parasite identification also varies among study sites but the same procedure was used in both samplings. Blood parasites were identified to genus and counted by microscopy or by molecular methods. The level of parasite identification and blood parasite lineage names can be obtained from column “Parasites” in S1 Table. No new sequences have been generated for this study. References with description of methods of sampling, identification and quantification are offered in S3 Table.

### Ethics statement

Permissions might have been requested for purposes others than sampling parasites in a target study wild population. Each researcher managed their own permission to obtain that data. All data used in this study comes from wild populations. Studies only collecting and quantifying ectoparasites usually do not require additional permits by ethical committees:

Coimbra, Portugal: Procedures complied with the ethical standards of the European and Portuguese guidelines for animal welfare (EU Directive 72 2010/63/EU and Portuguese Decree-Law no113/2013) and were approved by the Instituto da Conservação da Natureza e Florestas (ICNF).

Badajoz, Spain: Methods were evaluated and approved by the institutional Commission of Bioethics of the University of Extremadura (CBUE 49/2019)

Seville, Spain: All experimental procedures for project PGC2018–095704-B-I00 were approved by the CSIC Ethics committee and Animal Health authorities and complied with Spanish laws.

Granada, Spain: All procedures were conducted under license from the Consejería de Agricultura, Ganadería, Pesca y Desarrollo Sostenible of the Regional Government of Andalucía, Spain (reference DGPAG/SA/SIS). All applicable international, national, and/or institutional guidelines for the care and use of animals were followed.

Almeria, Spain: Permission authorized by the CSIC Ethics committee.

Valsain, Spain: The work was approved by Consejería de Medio Ambiente de la Comunidad de Madrid (approval ref. PROEX 088.7/22)

El Kala, Algeria: The advisory board of the laboratory of Environmental Biosurveillance gave approval for this work. The agreement of El-Kala National Park where the fieldwork was carried out was also obtained.

Corsica, France: Blue tit *C. caeruleus* captures and ringing were done under authorizations delivered by the Centre de Recherche sur la Biologie des Populations d’Oiseaux (CRBPO, Paris, permit for Anne Charmantier no. 1907).

Antwerp, Belgium: Procedures were formally approved by the Committee for Animal Experiments of the University of Antwerp, code ECD 2022−02.

Milovice forest, Czechia: Work was approved by the Committee for animal care and control and done under permits 50982/ENV/14–2961/630/14 and MZP/2019/630/1081 of the Ministry of the Environment.

Budapest, Hungary: This study received prior approval from the National Scientific Ethical Committee on Animal Experimentation and the Department of Environment and Nature Protection of the Hungarian Government Office.

Lomianki, Poland: The research followed ethical guidelines and was approved by the General Directorate of Environmental Protection and the Local Ethical Committee in Warsaw (permits no. 82/2012, 629/2014, 242/2020).

Kraghede (Denmark): Field sampling was conducted in agreement with the national Danish hunting law. Permit for capture and release of birds was obtained from the Danish Ministry of Agriculture.

Harjavalta, Finland: The ectoparasite samples from Pied flycatchers *F. hypoleuca* and Great tits *P. major* were collected under the license (VARELY/3622/2017) of the Centre for Economic Development, Transport and the Environment of Southwest Finland.

### Parasites

Following the method used by Møller et al. [22], we distinguished among four functional parasite groups based on their taxonomy and transmission dynamics: blood parasites, feather parasites, dipteran parasites, and non-dipteran parasites.

In our study, blood parasites were vector-borne endoparasites, and included apicomplexans (genera *Plasmodium*, *Haemoproteus*, *Leucocytozoon* spp. [25], and *Lankesterella* spp. [26,27]), kinetoplastids (*Trypanosoma* [28]), and the nematodes microfilariae [29].

Feather ectoparasites in our study were chewing lice (Insecta, Phthiraptera) and feather mites (Acari). They live in the plumage of the birds and are transmitted by contact or phoresis, and they are usually considered of low pathogenicity or even mutualist or commensal organisms in healthy hosts [30,31].

Dipteran ectoparasites include insects like louse flies (Hippoboscidae) and blowflies (Calliphoridae), which rely on their high autonomous mobility to reach host. In louse flies, adults feed on blood [28], while in bird blowflies, the larvae are haematophagous [32].

Non-dipteran parasites included ticks (Ixodidae) and other blood-sucking mites (Dermanyssidae), as well as fleas (Siphonaptera, Ceratophyllidae), whose autonomous mobility is lower than that of dipterans. Only adult fleas are hematophagous, while several or all developmental stages of the studied mites and ticks feed on host blood [28,33,34].

When more than one taxon of parasite within any of these groups was investigated in a target host species and population, we estimated mean values, which were used for subsequent analyses (same approach as in [22]).

We requested information on prevalence (the proportion of adult birds or nests harboring a given parasite) and the mean intensity of parasites (mean number of parasites per infested bird host or infested bird host nest). Since our statistical analyses dealt with between-year differences in parasitism prevalence and intensity, and those differences are independent of the sampling methods, estimated differences for different host populations would be comparable to each other.

### Temperature trends

We used the package ‘NicheMapR’ 3.2.1 to extract local temperature data from every sampling coordinate at a 10 arc minutes of resolution [35] where we set the month and year of sampling as a download timeframe. We downloaded air temperatures (°C) at 1 m over ground level (TAREF in [35]). Thus, for every sampling coordinate, and given month and year (when the peak of the hatching date occurred at each population), we obtained a single monthly average of daily measures for each local climate variable. Change in temperature between the months of sampling was estimated as the temperature in 2021−22 minus the temperature in 2010−11 (or the corresponding date of second and first sampling). Although the interval between the two study years is almost invariably 11 years, we divided the estimated temperature differences by the interval between the two years just to reach values comparable to those in Møller et al. [22]. This change in temperature over time (ºC/year) is referred to as the change in temperature between study years throughout the remainder of this paper.

### Life history and population density of hosts

We requested all participants to record the laying date of the first egg, clutch size, number of fledglings and body condition of the parents estimated as body mass measured during the period of the parents feeding the young for each host individual included in this study. We also requested a local estimate of population density of hosts (measured at each locality following the same protocol, that is, for nest box studies, the proportion of nest boxes occupied or the total number of occupied nest boxes, and for open nesting species the number of individual birds captured, colony size, or, in a few cases, population density). The entire dataset and the average values used in statistical analysis are reported in the supplementary material (S1 and S2 Tables).

### Statistical analyses

Brood size, host population density, and parasite prevalence and intensity were log_10_ transformed to achieve approximately normal distribution (Kolmogorov-Smirnov tests for continuous variables, P > 0.05), while distribution of raw data of laying date (1 = 1^st^ of April), clutch size, latitude, temperature change (ºC) per year among study year approximately showed a Gaussian shape (Kolmogorov-Smirnov tests for continuous variables, P > 0.2). Moreover, the average body mass of adult birds, as well as between-years differences in body mass, greatly varied among the considered species (i.e., body mass of *C. garrulus* is almost one order of magnitude heavier than that of other species). Thus, we have standardized body mass of each species within each population by dividing estimated body mass in each study years by the average of body mass of the two study years (e.g., Standardized Body Condition (Year1) = Body mass (Year1) / ((Body mass (Year1) + Body mass (Year2)) /2)), which approach a normal distribution (Kolmogorov-Smirnov tests for continuous variables, P > 0.2).

The changes in host population parameters, parasite intensity and prevalence between years were tested by Repeated Measures ANOVAs with laying date, clutch size, brood size, body condition of adult birds, population density, parasite intensity and parasite prevalence estimated for the two study periods as within-subject (repeated measures). Host population identity, host identity, identity of parasite functional group, latitude, and temperature change per year (ºC/years) were used as between subject factors. All analyses were performed in separate models (30 models in total) in order to avoid inclusion of more than one interaction term of between and within subject factors (i.e., repeated measures).

The effects of parasitism on characteristics of host populations were also explored using Repeated Measures ANOVAs with laying date, clutch size, brood size, body condition of adult birds and population density estimated for the two study periods as within subject factors (repeated measures), and change in parasite intensity and prevalence as between subject factors. Parasite functional group, latitude and temperature change (ºC/years) were also included in the models as additional between subject factors to statistically control for the effect of these factors on changes in host population characteristics. A significant interaction between the repeated measure factor and differences in parasitism would suggest a parasite-related change in host population characteristics. The effects of interactions by parasite functional group and intensity or prevalence of parasitism were far from statistical significance (P > 0.454) and were not included in the statistical models.

All analyses were weighted by the log_10_ of sample size (the sum of individuals or nests sampled in the two study periods with information on parasite prevalence) to ensure that all individual observations contribute relative to their precision based on sample size [36,37]. All analyses were performed with Statistica 13 [38]. Values reported are means of transformed values (SD or 95% CI).

## Results

The mean change in temperature per year across the 17 data sets (i.e., bird species per location) was + 0.003 ºC (SD = 0.161, Range = −0.287 - + 0.211) (S2 Table). The number of years between the first and the last study year was on average 11 years (SD = 1.98). 62 host-parasite interactions were studied that were grouped in 15 blood parasites, 4 non-dipteran parasites, 6 dipteran parasites and 3 feather parasites (Table 1).

**Table 1:** Repeated Measures ANOVAs with parasite intensity and parasite prevalence in the two separate study years as within subject factors (Repeated Measures (RM)), and locality, parasite functional groups (only for models considering all groups of parasites together), latitude interval in years between both periods and temperature change (ºC/year) as between subject factors. Each effect was estimated in separate models. P-values smaller than 0.05 are shown in bold.

	Parasite intensity			Parasite prevalence		
	F	df	P	F	df	P
**Temporal changes (RM)**	4.02	1, 18	0.060	0.07	1, 23	0.792
RM * Locality	1.69	9, 9	0.224	1.09	13, 10	0.457
RM * Parasite functional group	0.75	3, 15	0.541	2.30	3, 20	0.108
RM * Latitude	**5.58**	**1, 17**	**0.030**	0.01	1, 22	0.930
RM * Intervals in Years	0.44	1, 17	0.518	0.19	1, 22	0.670
RM * Temperature change (ºC/Y)	0.52	1, 15	0.483	0.34	1, 20	0.566

### Temporal change in host populations

**Fig 2:**
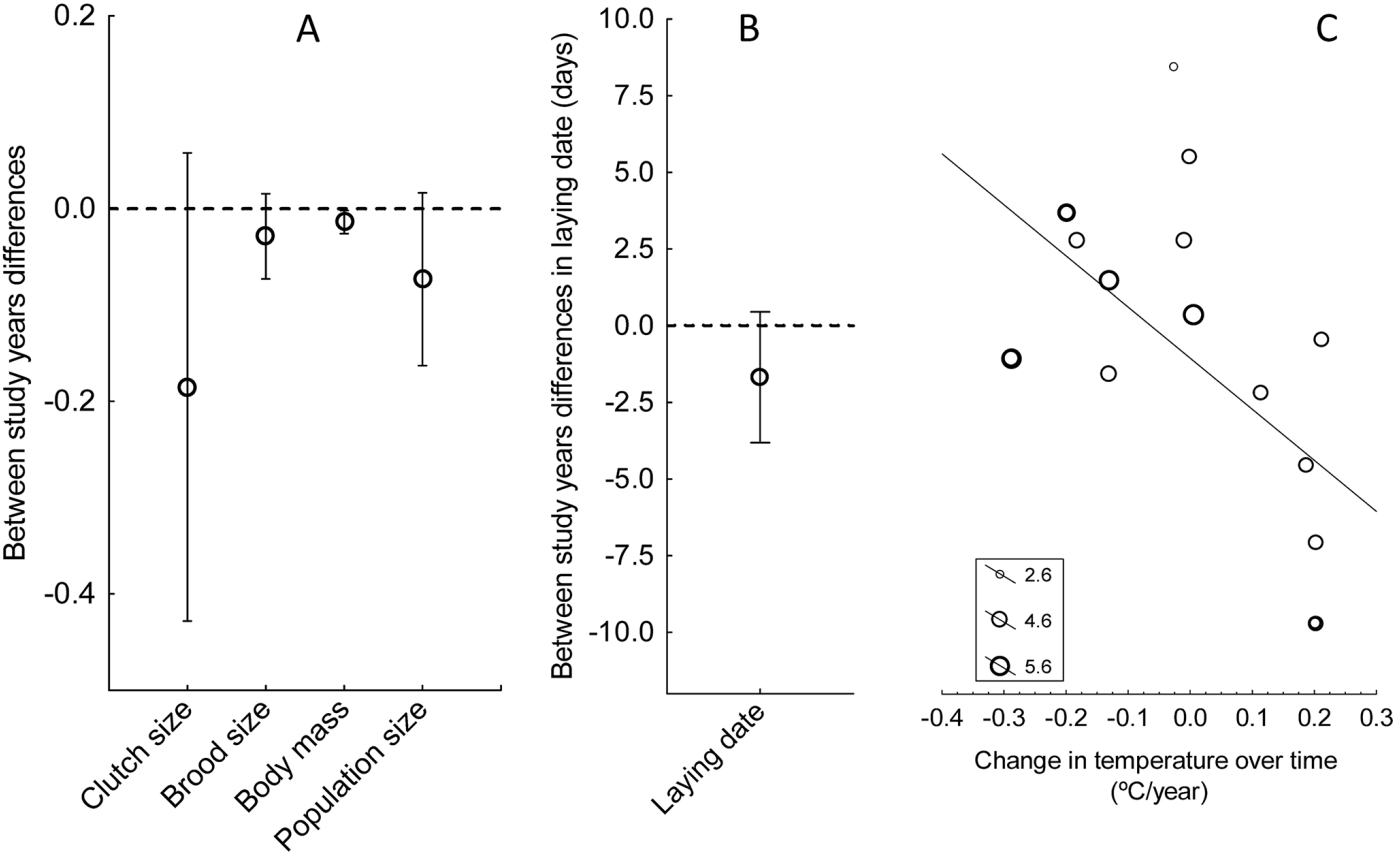
Temporal changes in clutch size, brood size (log_10_ transformed), host population size (log_10_ transformed), relative body mass to the between years average value (body mass) (A) and laying date (B) of hosts between the last (2021-2022) and the previous study periods (2005-2015). Plots show means ± 95% CI. The laying date and temperature change between study years were negatively associated (C). In C, dot size is proportional to log_10_-transformed sample size of each of the studied population, while the line is the regression line.

Host populations on average tended to start laying eggs earlier, laid smaller clutches and raised smaller broods during 2021 than in the previous sampling year (second sampling of the Møller et al. [22] study or the previous for new studies included in this work) although differences between years were not statistically significant (all P > 0.07; Table 2; Fig 2). Host population size and host body condition tended to decrease in recent years (Table 2; Fig 2). Moreover, none of these trends depended on host species or population identity, latitude, or intervals in years, but the temperature change experienced by each population was associated negatively with the change in laying date (Table 2). Laying dates advanced in populations with increased temperature and delayed in those with reduced temperature between years (Fig 2).

**Table 2:** Repeated measures ANOVAs with laying date, clutch size, brood size, body condition and host population density of hosts in two separate study years as within subjects’ factors (Repeated Measures (RM)) and locality identity, host identity, latitude, interval in years and temperature change (ºC/year) as between subjects’ factors. Each effect was estimated in separate models. P-values smaller than 0.05 are shown in bold.

	Laying Date			Clutch size			Brood size			Body mass			Population density		
	F	df	P	F	df	P	F	df	P	F	df	P	F	df	P
**Temporal changes (RM)**	1.01	1, 14	0.332	3.05	1, 13	0.104	3.76	1, 13	0.074	3.19	1, 13	0.097	1.10	1, 16	0.309
RM * Locality	2.60	11, 3	0.233	0.47	10, 3	0.831	0.70	10, 3	0.707	0.26	10, 3	0.954	1.68	13, 3	0.370
RM * Host identity	0.47	6, 8	0.813	1.14	6, 7	0.429	2.83	6, 7	0.099	0.96	7, 6	0.530	2.72	8, 8	0.089
RM * Latitude	0.61	1, 13	0.449	0.12	1, 12	0.732	0.00	1, 12	0.952	1.64	1, 12	0.224	0.14	1, 15	0.715
RM * Intervals **in Years**	0.50	1, 13	0.491	1.39	1,12	0.261	1.75	1, 12	0.210	0.61	1, 12	0.451	0.79	1, 15	0.387
RM * Temperature change (ºC/Y)	**5.53**	**1, 12**	**0.037**	1.25	1, 11	0.288	0.71	1, 11	0.417	0.33	1, 12	0.575	0.04	1, 14	0.839

### Temporal change in parasite populations

**Fig 3:**
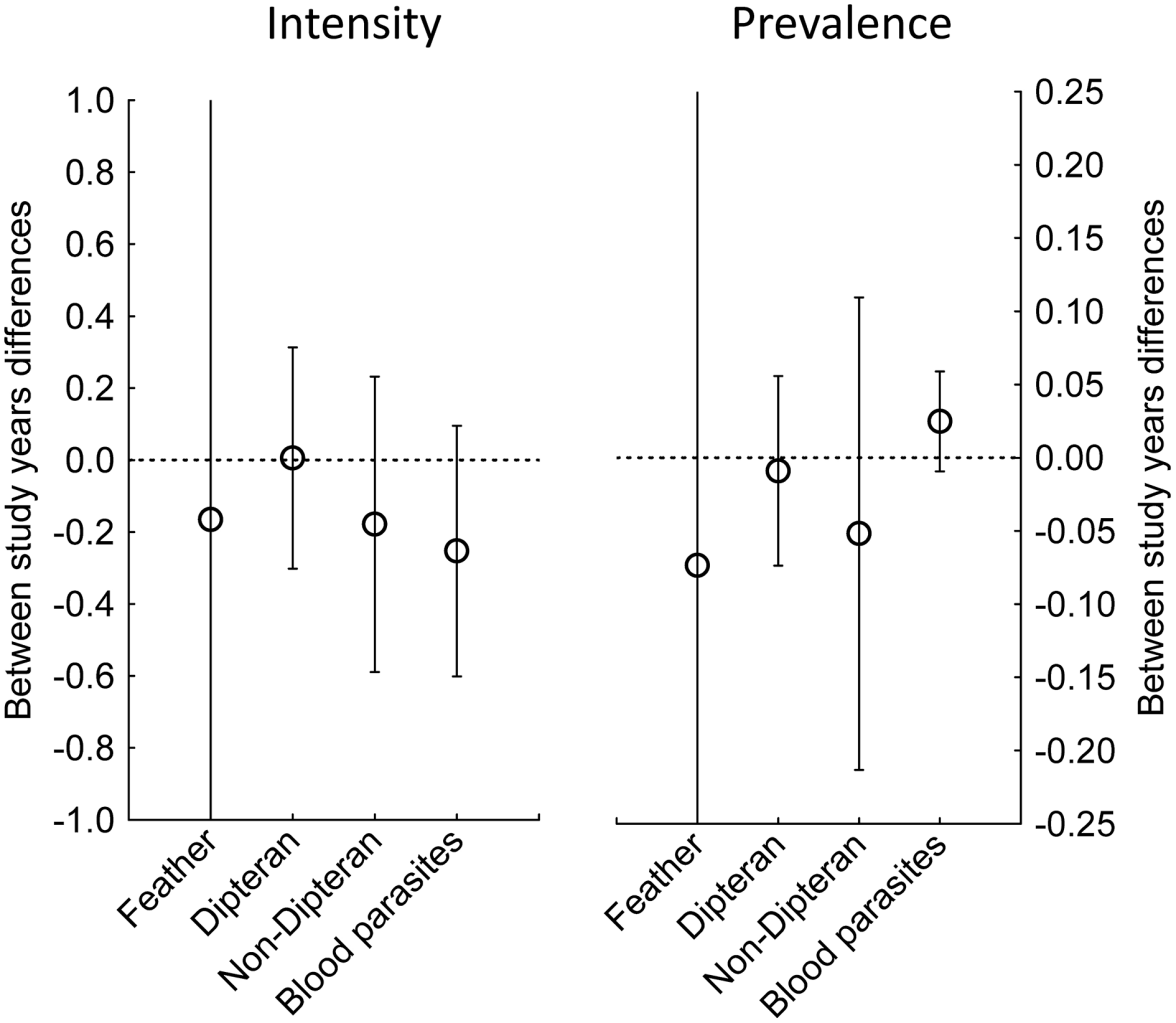
Temporal changes in intensity and prevalence of the four parasite functional groups analyzed between the last (2021−2022) and the previous (2005−15) study periods. Plots show means ± 95% CI.

**Fig 4:**
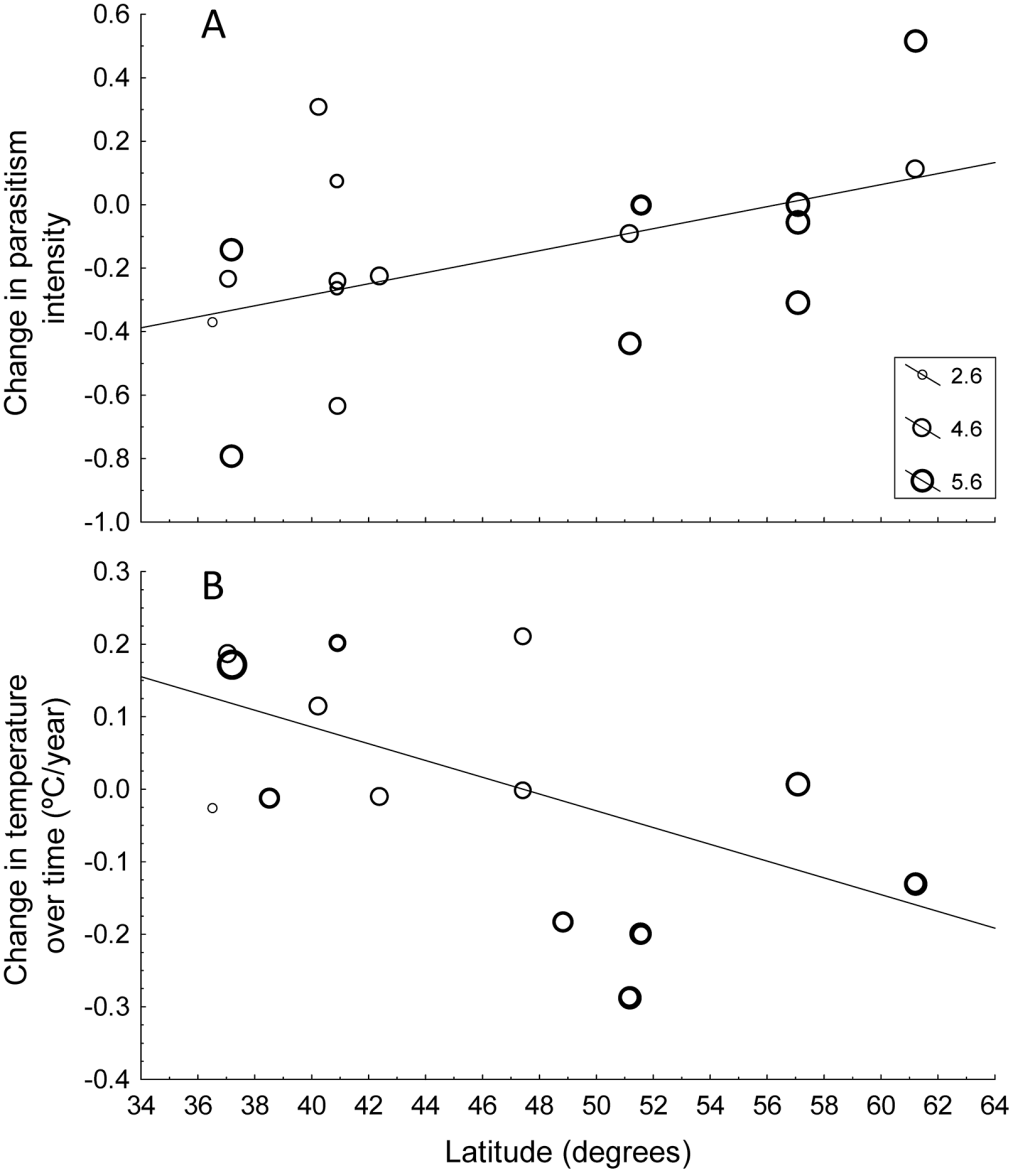
Temporal change in parasite intensity (A) and temperature (B) between study periods with latitude (degrees). A shows the detected effect of latitude on change in parasitism intensity and, thus, the 19 data points refer to the host species with information for one or more types of parasites and populations. B shows values for each of the 14 study populations with information on temperature change. The size of the dots is proportional to log_10_-transformed sample sizes, while the line is the regression line.

We detected 11 gains, that is parasites not detected in the first sampling year that were detected in the second year, all among blood parasites, and 0 losses of parasites among studies. All parasite groups, except blood parasites, tended to be less abundant and less prevalent in 2021−22 than in the previous sampling season; however, these trends did not reach statistical significance (Table 1; Fig 3). At higher latitudes the intensity of parasites decreased less than at lower latitudes (Table 1; Fig 4A). In addition, the temperature change was also associated with latitude (r = 0.60, n = 16, P = 0.014; Fig 4B), with a higher increase in temperature between the two sampling seasons in southern than in northern areas (Fig 4B). Importantly, there were no differences in the distribution of the studied parasite types along the latitudinal gradient (F_3,20_ = 1.76, P = 0.19).

### Temporal change of the relationships between host and parasite populations

Differences in clutch size, brood size, host body condition and host population density between the two study periods were not associated with differences in parasitism intensity or prevalence, nor did they depend on the considered parasite taxa (Table 3). That was the case after controlling for the effects of latitude and temperature changes between study periods (Table 3).

**Table 3:** Repeated measures ANOVAs with laying date, clutch size, brood size, body condition and host population density in two separate study periods as within subjects’ factors (Repeated Measures (RM)) and interactions between the repeated measure and latitude, temperature change, variation in parasitism (i.e., temporal change in intensity and prevalence of parasites) and identity of parasite group (parasite ID) as between subject factors. Beta-values of the association between differences in laying date, clutch size, brood size, body condition and host population density as independent continuous factors (latitude, temperature change and variation in parasitism (i.e., change in intensity and prevalence of parasites)) are also shown. Separate analyses for data sets including parasite intensity or prevalence in the first and the second year are shown. Associated P-values smaller than 0.05 are shown in bold.

	Temporal changes (RM)			RM * Latitude				RM * Temperature change				RM * Parasitism				RM * Parasitism ID		
	F	df	P	Beta	F	df	P	Beta	F	df	P	Beta	F	df	P	F	df	P
**Parasite intensities**																		
***Mean values***																		
Laying date	0.19	1, 10	0.675	−0.219	0.41	1, 10	0.536	**−0.681**	**5.96**	**1, 10**	**0.035**	0.215	0.73	1, 10	0.413	0.50	3, 10	0.690
Clutch size	0.67	1, 10	0.432	0.291	0.60	1, 10	0.458	**0.773**	**6.33**	**1, 10**	**0.031**	−0.021	0.01	1, 10	0.941	0.15	3, 10	0.926
Brood size	3.10	1, 10	0.109	0.556	2.90	1, 10	0.119	**0.842**	**10.05**	**1, 10**	**0.010**	−0.188	0.62	1, 10	0.450	0.49	3, 10	0.699
Body condition	0.22	1, 9	0.654	0.159	0.09	1, 9	0.775	0.265	0.40	1, 9	0.543	−0.094	0.07	1, 9	0.799	0.24	3,10	0.868
Population density	**5.04**	**1, 10**	**0.049**	**−0.886**	**5.89**	**1, 10**	**0.036**	−0.326	1.20	1, 10	0.299	0.325	1.48	1, 10	0.252	0.56	3, 10	0.651
**Parasite Prevalence**																		
***Mean values***																		
Laying date	0.08	1, 13	0.782	0.034	0.01	1, 13	0.911	**−0.576**	**4.79**	**1, 13**	**0.047**	0.255	0.92	1, 13	0.355	0.92	3, 13	0.856
Clutch size	0.11	1, 12	0.743	0.089	0.07	1, 12	0.801	0.556	3.22	1, 12	0.098	0.109	0.13	1, 12	0.724	0.26	3, 12	0.856
Brood size	1.53	1, 12	0.250	0.363	1.46	1, 12	0.250	**0.711**	**6.98**	**1, 12**	**0.021**	0.169	0.41	1, 12	0.532	0.86	3, 12	0.489
Body condition	0.00	1, 13	0.948	−0.147	0.11	1, 13	0.743	0.005	0.00	1, 13	0.999	−0.215	0.45	1, 13	0.515	0.14	3, 13	0.935
Population density	0.15	1, 15	0.705	−0.196	0.31	1, 15	0.587	−0.090	0.09	1, 15	0.768	0.431	2.14	1, 15	0.164	0.21	3, 15	0.887

## Discussion

In this study, we found that parasite intensity differentially decreased at lower latitudes (Fig 4A) when comparing the two study periods. Although mean temperature increased between the two sampling years when all localities were considered, at higher latitudes temperature generally decreased between the two study years (Fig 4B). In other words, a greater increase in temperature appears to be associated with a greater reduction in the intensity of parasitism. However, one limitation of our study is the fact that we only studied the change in temperature between two years and, of course, temperature can change in different ways at different localities during the different periods elapsed between the sampling years. Here we only compare the change in temperature during the breeding months of each bird population between two years and divide the difference by the number of years elapsed due to the differences in time between sampling years for different studies. Few studies have shown latitudinal changes in the incidence of parasites in birds (reviewed in [19]). The existence of latitudinal gradients in parasitic-species richness or prevalence could be the simple consequence of similar gradients occurring in their host species diversity [14], but climatic factors can also have an influence [13] or distort the gradient [19]. Thus, the change of parasitism intensity with latitude and the corresponding change in temperature with latitude suggest that climatic change can potentially affect host-parasite interactions over time and potentially alter latitudinal gradients, with higher parasite incidence at lower latitudes. This pattern was not observed in the previous work comparing changes in bird populations sampled in 2010 and approximately ten years earlier [22]. By repeating the same design ten years later, we found a latitudinal effect on the change in temperature and parasite intensity suggesting that conditions for host-parasite interactions have changed over time in the same geographical area (Fig 4).

Many parasites depend on the availability of water or humidity in order to complete their life cycles. For example, most blood parasites are transmitted by vectors that need water sources or certain levels of humidity for larvae development (e.g., mosquitoes and other blood-sucking insects [39–41]). Arthropods in general, including ectoparasites such as ticks or fleas, are also affected by relative humidity [42–44]. In this sense, it would be expected that a greater increase in temperature (and droughts) in southern areas had reduced the presence of water sources or relative humidity necessary for these parasites. If that were the case, the observed increase in temperatures in southern Europe may be negatively affecting many parasite species, while the arrival of milder climates in northern regions may either positively influence parasite intensity or be less detrimental to them.

Experimental studies have shown that increases in humidity and temperature can negatively affect parasite populations [7,8]. However, long-term studies seem to indicate that parasites have so far been able to adapt to changes in climate, and even are increasing their populations as for example, nest-dwelling ectoparasites [3,5,6], or viruses [45]. Although our study found that intensity and prevalence of parasites tended to decrease, this trend did not apply to all types of parasites, as the prevalence of blood parasites tended to increase as it occurs for several haemosporidians (genera *Plasmodium*, *Haemoproteus*, *Leucocytozoon*) in several host species and localities (Fig 3; S1 and S2 Tables). Our study also shows that prevalence and intensity of Diptera did not change between years (Fig 3). Given that many Diptera species act as vectors of blood parasites (like Haemosporidia or microfilariae) we expected that prevalence of the latter would not change either although many other factors can also play a role in blood parasite prevalence. Among the dipterans considered in this study, only louse flies act as vectors of some of the blood parasites studied such as some haemosporidians and trypanosomes. However, we found an increase in the prevalence of blood parasites between years but a decrease in their intensity. In addition, 11 gains in the group of blood parasites were detected across all host populations (S1 Table). The increased prevalence could be related to the abundance of particular types of vectors [46], while the decrease in blood parasite intensity may be more related to the immunocompetence of hosts [47] or to different phenology of vector occurrence leading to sampling bias due to different transmission periods [48]. Whatever the mechanism, the effect of climate on vectors may affect the prevalence and intensity of blood parasites in vertebrate hosts, but such effects may differ between vectors.

In addition, temperature increases could lead to droughts, concentrating host and vector around water sources, therefore increasing parasite circulation [49]. In any case, these trends for parasite intensity and prevalence are not significant and therefore must be verified with subsequent studies.

Our analyses also showed a temporal non-significant reduction in reproductive success (clutch size and brood size) and body mass in bird populations across Europe, as well as an advancement in the breeding phenology of birds in populations where temperature increased between sampling years (Fig 2). Some of these trends have been reported previously in studies for particular host populations [6,50–52]. In general, many bird species have advanced their egg laying with time [53–63]. This is due to an attempt by the birds to match the changes in the phenology of their main prey, e.g., caterpillars [58,63–68], which in turn follow changes associated with temperature [69] and vegetation [63,70]. Although temperature is important, other associated factors, such as precipitation, may also play a role and droughts, which are becoming increasingly common, particularly in southern Europe, and have significant effects on bird populations [71].

In conclusion, our study reveals different trends in the between-years changes of both parasites and their avian host populations over time than those reported by Møller et al. [22]. For instance, we did not detect a consistent trend in the intensity of parasitism across years (Fig 3), but instead found among-study-years variations in both parasitism and temperature, influenced by latitude (Fig 4). In addition, while their study reported delayed laying date, and increases in body condition and brood size, our results showed a tendency for an advancement in laying date and decreases in body mass and brood size between years (Fig 2). Moreover, since variation in parasitism across years is associated with latitude, and temperature change across years is also negatively related with latitude, it is possible that variation in parasitism intensity was mediated by the effects of latitude in temperature change over time. (Fig 4). However, the detected clear latitudinal effects in the magnitude of the change in parasite populations reported here may also well be associated with the detected positive effect of latitude in temperature change and require a more detailed analysis of the responses of different parasite groups to long-term changes in climate.

## Supporting information

S1 Table Raw data on host, parasites and localities used in this study. (XLSX)

S2 Table Data grouped by parasites functional groups as used in this study. (XLSX)

S3 Table List of references on methods used by each study included in this work. (XLSX)
